# An under-represented and underserved population in trials: methodological, structural, and systemic barriers to the inclusion of adults lacking capacity to consent

**DOI:** 10.1186/s13063-020-04406-y

**Published:** 2020-05-29

**Authors:** Victoria Shepherd

**Affiliations:** Centre for Trials Research, Cardiff University, Cardiff, UK

**Keywords:** Informed consent, Mental capacity, Randomised controlled trials, Inclusion, Underserved populations

## Abstract

**Background:**

There is increasing international recognition that populations included in trials should adequately represent the population treated in clinical practice; however, adults who lack the capacity to provide informed consent are frequently excluded from trials. Addressing the under-representation of groups such as those with impaired capacity to consent is essential to develop effective interventions and provide these groups with the opportunity to benefit from evidence-based care. While the spotlight has been on ensuring only appropriate and justifiable exclusion criteria are used in trials, barriers to the inclusion of adults lacking capacity are multifactorial and complex, and addressing their under-representation will require more than merely widening eligibility criteria. This commentary draws on the literature exploring the inclusion of adults lacking the capacity to consent in research and a number of recent studies to describe the methodological, structural, and systemic factors that have been identified.

**Main text:**

A number of potentially modifiable factors contributing to the under-representation of adults lacking the capacity to consent in trials have been identified. In addition to restrictive eligibility criteria, methodological issues include developing appropriate interventions and outcome measures for populations with impaired capacity. Structurally determined factors include the resource-intensive nature of these trials, the requirement for more appropriate research infrastructure, and a lack of interventions to inform and support proxy decision-makers. Systemic factors include the complexities of the legal frameworks, the challenges of ethical review processes, and paternalistic attitudes towards protecting adults with incapacity from the perceived harms of research.

**Conclusions:**

Measures needed to address under-representation include greater scrutiny of exclusion criteria by those reviewing study proposals, providing education and training for personnel who design, conduct, and review research, ensuring greater consistency in the reviews undertaken by research ethics committees, and extending processes for advance planning to include prospectively appointing a proxy for research and documenting preferences about research participation. Negative societal and professional attitudes towards the inclusion of adults with impaired capacity in research should also be addressed, and the development of trials that are more person-centred should be encouraged. Further work to conceptualise under-representation in trials for such populations may also be helpful.

## Background

Recent efforts to reduce health inequities have failed to address the significant disparities that persist for some population groups in accessing high-quality care. One barrier to achieving health equity is the inadequate recruitment of under-represented and underserved populations in research [1]. These research inequities contribute, in part, to the lack of evidence for providing effective care and treatment, resulting in evidence-biased care for some groups [2]. There is increasing international recognition that populations included in clinical trials should adequately represent the population treated in clinical practice [3], a position reinforced in the upcoming Clinical Trials Regulation No. 536/2014 that states that the participants in a clinical trial should represent the population groups that are likely to use the medicinal product investigated in the clinical trial [4]. The lack of representation of certain groups in trial populations brings the external validity (the extent to which the results can be generalised to other circumstances or populations) [5] of trials into question, which is even more important when these groups are systematically excluded from those trials [6, 7]. This has led to major research funders, such as the UK National Institute for Health Research (NIHR), to lead projects to address the challenges of carrying out health research with under-represented groups (e.g. NIHR INCLUDE [8]) and to introduce reviews of the suitability of, and justification for, inclusion and exclusion criteria as part of their review processes [9].

Research involving vulnerable groups, such as adults lacking mental capacity, raises many ethical and legal issues—particularly with respect to informed consent for them to be included in a project [10, 11]. As a result, recruitment of vulnerable groups to research is a complex process [12, 13] and the exclusion of people with cognitive impairment from research is widespread [6, 7]. The exclusion of those who lack the capacity to consent from participating in research has been highlighted as a concern in populations where the prevalence of impaired capacity is high [14] and where less is known about what works most effectively to improve care [15]. For example, the Learning Disabilities Mortality Review programme found that people with learning disabilities die, on average, 15–20 years earlier than the general population, with many deaths being potentially amenable to health care interventions [16]. People living with a learning disability have higher rates of long-term health conditions, such as cardiovascular disease, diabetes and mental illness, and are more likely to be admitted to hospital as an emergency [16]. However, there is a lack of an evidence base for many of the interventions and treatments provided to people with learning disabilities [17]. Previous studies have found that over 90% of randomised controlled trials are designed to exclude people with a learning disability [6]. A recent review of studies on the NIHR portfolio found that 60% of the studies excluded learning disability groups, none of the studies investigating pneumonia or sepsis included learning disability groups despite being a key contributor to premature deaths, and only 1.4% of all studies were specifically related to learning disabilities [18]. Similar exclusions of people with cognitive impairment are seen in other areas of research such as geriatrics [7], rehabilitation interventions following hip fracture [19], peri-operative medicine [20], trauma [21] and neurological research [22]. Under-recruitment to research into conditions such as dementia is one of the key challenges to advancing understanding of these conditions and improving the care and treatment of those who live with these conditions [23].

With growing evidence of the widespread exclusion of adults lacking capacity from clinical trials and other research, there have been calls for a review of the ethical barriers to the inclusion of these populations, and for greater active involvement of research funding organisations to scrutinise the justifications behind this exclusion [18]. However, this issue is more complex than might be first thought. The recruitment of adults with impaired capacity is situated within a complex system of factors [24], and the differences between legal jurisdictions and how different types of research are governed within these jurisdictions adds further complexities [2]. Research governance and funder oversight that focuses on ensuring that appropriate eligibility criteria are used will not be effective in increasing inclusivity in trials for adults who lack capacity unless the wider barriers to inclusion are recognised and addressed.

The exclusion of adults who lack capacity has been the subject of two recent publications in *Trials.* In the first, we presented data on the concerningly low number of trials designed to include adults lacking capacity identified through an analysis of clinical trials in conditions commonly associated with cognitive impairment that were completed in the previous 3 years [25]. The second was a response to our study, in which Griffiths et al. detailed the practical challenges encountered in developing their trial involving hard-to-reach groups of people living with dementia in the community, and the potential implications for being able to include those with impaired capacity [26]—arguably the hardest of all the groups to reach. The barriers they described included gaining a favourable ethical opinion, designing recruitment pathways that ensure people living with dementia have the opportunity to participate in circumstances when their cognitive disability might impair their ability to read or understand an invitation letter, and the challenges of identifying consultees to be involved in decisions about their participation [26].

This commentary discusses and expands on some of the issues raised by Griffiths et al., drawing on a non-systematic review of the literature, experience of conducting trials with populations with impaired capacity [13, 27, 28], and recent findings from a series of studies exploring the involvement of adults who lack the capacity to consent [25, 29–32]. The aim is to articulate for the first time some of the wider methodological, structural, and systemic barriers to the inclusion of adults lacking capacity as a first step towards identifying and addressing some of the potentially modifiable factors. This is presented from the perspective of trials conducted in the UK, but will have implications and relevance for research conducted in other legal jurisdictions.

## Barriers to inclusion in research

### Restrictive eligibility criteria

Eligibility criteria are used in trials to recruit participants that are representative of the patient populations who will ultimately receive the medication or intervention in clinical practice, and to ensure the safety and protection of participants [33]. However, some trials use very restrictive eligibility criteria that exclude groups without valid reasons resulting in research samples that do not represent the diversity, symptom complexity or daily challenges of the clinical population [22]. This negatively impacts on the external validity of the trial [33]. Excluding patients with cognitive impairments who are unable to provide consent is common, although justification is rarely provided or discussed as a potential limitation of the trial findings [6, 7, 22]. While eligibility is an essential component of trial design, arbitrary exclusion of large numbers of the clinical population restricts the pool of potential participants, affects the generalisability of the results and, as these excluded individuals are disproportionately from vulnerable populations, raises ethical concerns [22]. The lack of inclusion of adults who lack capacity in trials that are directly relevant to them should be carefully scrutinised by funders and those determining the scientific merit of proposed trials and, where they are excluded, an evidence-informed justification for their exclusion should be explicitly stated [19].

### Complexities of legal frameworks

The legal frameworks that govern research involving adults who lack capacity to consent varies between legal jurisdictions, even within countries, and in different types of trials depending on whether the intervention is an investigational medicinal product or not [2]. These complexities impact on the ability to conduct studies involving adults lacking capacity. For example, two separate regulatory regimes govern research involving adults who lack capacity to consent in England and Wales, where the Mental Capacity Act 2005 governs how adults lacking capacity can be involved in research considered invasive [34]; however, clinical trials of investigational medicinal products (CTIMPS) are separately regulated by the Medicines for Human Use (Clinical Trials) Regulations 2004 [35]. There are significant differences between these regulatory frameworks, including the level of risk permitted, who acts as proxy decision maker, how much information is provided to the person lacking capacity, and whether or not they retain the power of veto [2]. The impact of different legal arrangements was demonstrated in our recent clinical trial conducted in care homes in which we found that the process for setting up a CTIMP in care homes was more complex and time consuming than the process for setting up an observational study in the same setting or setting up CTIMPs in other health care settings [13]. Other jurisdictions within the UK (Scotland and Northern Ireland) have different legislation governing research involving adults with incapacity, and there is a similarly problematic ‘patchwork of legislation’ within Australia [36], the US [37] and the EU [38]. One EU-wide study to investigate the epidemiology and genetics of sepsis in critical care patients found that the outcomes when seeking ethical approval differed widely between countries because of differences in national legislation, and within countries because of interpretation of the ethics of conducting research with people lacking capacity [39].

A number of studies have found a widespread lack of knowledge and understanding about the legal frameworks by health care professionals and researchers, including in the UK [29], US [40], and Canada [41], leading to calls for greater education and awareness about the legal frameworks. The UK survey I led found that health and social care professionals caring for populations who lack capacity did not understand that decision-making processes about research participation differed from usual care and treatment decisions (research decisions are based on the person’s ‘presumed will’ and not what was in their best interests), differed between different types of research (a legal representative provides consent for CTIMPs whereas a consultee merely provides advice to researchers for other types of studies), and who could act as a personal legal representative or consultee (family members regardless of any legal arrangements such as having Power of Attorney, or someone such as themselves acting in a professional capacity in the absence of a family member or friend) [29]. Our recent research found similar misconceptions and misunderstandings were evident in study documents that had been designed by trial teams and undergone review by research ethics committees (RECs) and received a favourable opinion [30]. A number of trial information sheets contained inaccurate interpretations of the legal frameworks, including who could act as proxy decision-maker (e.g. holder of Power of Attorney), the basis for their decision (e.g. best interests), the role title and basis (e.g. consultee providing consent rather than advice), and conflated professionals’ clinical and representation roles [30]. We concluded that there is a need for education and training interventions to inform and support those who design and conduct research studies that include adults lacking capacity, as well as RECs responsible for the ethical review of such studies [30].

### Ethical review processes

Research ethics has historically been concerned with the protection of ‘vulnerable groups’ from participation in research [42]. By contrast, there has been much less concern about ensuring that these groups are not denied the opportunity to receive the potential benefits of participation that are available to others, which may lead to harm for these groups through being denied the opportunity to benefit from scientific advances and receive evidence-based care. The recent update of international ethics guidelines (from the Council for International Organizations of Medical Sciences), where there is now a need to justify exclusion [43], has been seen as marking a paradigm shift from overprotection to inclusion [44]. However, this is yet to be reflected in the legal frameworks, and many researchers’ experiences of ethical review processes demonstrate the challenges of justifying the inclusion of adults who lack capacity in trials and other studies in practice.

The letter by Griffiths et al. published recently in *Trials* highlighted the challenges that studies designed to include adults who lack capacity encounter when seeking a favourable opinion from an REC [26]. The authors described a lack of awareness from the REC of the importance of including people who lack capacity in the development phase of a complex intervention (in this case a primary care-based support intervention for people living with dementia) to ensure that the intervention was appropriately designed for those who may benefit most from the intervention [26]. They called for improvements in the consistency of interpretation and advice from RECs so that hard-to-reach groups are enabled to take part in intervention development research [26]. There are also published accounts of a lack of consistency when studies are reviewed by National Health Service RECs, including how the requirements of the Mental Capacity Act 2005 are interpreted (e.g. whether the research is connected to an impairing condition or not), ambiguities about who can act as a nominated consultee (if a paid carer is permissible or not), and the ‘grey area’ of when treatment being evaluated is needed in a timely manner but is not an emergency [45]. A 2009 study reviewed REC reviews of studies involving adults who lacked capacity and found inconsistency in the interpretation of legal requirements, the use of incorrect terms, and the potential to exclude people who were eligible to be consulted about the involvement of adults who lack capacity [46]. The authors called for improved clarity, explicitness and accuracy when submitting and reviewing applications for ethical review of research in this area [46]. Our recent content analysis found similar issues with study documents that had undergone review by RECs and received a favourable opinion [30], suggesting that little has changed in the intervening decade and that the need for greater clarity and accuracy remains. While the Health Research Authority, which oversees RECs in the UK, are taking measures to address inconsistency in ethical reviews [47] this does not specifically apply to research involving adults lacking capacity.

### Challenges of proxy decision making

For adults who lack the capacity to consent to research, an alternative proxy or surrogate decision-maker is involved in the decision about their participation. The legal provisions for who can act as a proxy differ between legal jurisdictions and the type of research involved, including whether it is emergency research [2]. This presents a number of challenges, including procedures for assessing decision-making capacity, identifying and approaching proxies, and proxy decision-making processes. Trials involving adults lacking capacity are necessarily resource-intensive, given the skills and time required to: identify potential participants who are unlikely to be recruited through traditional methods such as responding to advertisements; build a relationship with the potential participant; determine eligibility and provide tailored information about the trial; sensitively conduct capacity assessments; identify suitable proxies who are able and willing to be involved; provide them with information about the trial; and seek their consent or agreement. These vital activities will require additional resources, recruitment periods, and skilled personnel that are likely to result in more costly and lengthy trials than those solely recruiting adults with the capacity to consent. Processes for recruiting adults who lack capacity are more complex and time consuming, and often occur outside established and traditional trial settings [27, 28]. Research infrastructure that supports trials and other research routinely conducted in health care settings may not be appropriate for trials that include populations where capacity is impaired in health and social care settings without additional training and resources. This may be additionally so for emergency research where recruitment is time critical, and models of consent such as deferred consent require ongoing capacity assessments and approaching participants and/or proxies for consent at later time points. Research funders should bear these additional requirements in mind when reviewing funding applications for trials that include adults lacking capacity.

Improving informed consent processes for clinical trials has been the focus of much attention in recent times, with numerous interventions to improve the provision of information [48], participant understanding [49], and decision making about participation [50]. Despite the additional complexity of proxy decisions about participation made on behalf of an adult who lacks capacity, and how the person’s preferences and wishes can best be respected, there has been little research in this area. Our research has identified the emotional and decisional burdens experienced by proxies making decisions about research [31], and developed the first intervention to support family members acting as proxies [24]. However, more research is needed to explore the experiences and support needs of family members and others acting as proxies (e.g. health care professionals in the absence of family members) and those who recruit adults lacking capacity in different settings. Extending the legal provisions to enable the prospective appointment of a research proxy prior to a loss of capacity, and a formal process for documenting preferences about participating in trials in the event of a loss of capacity, may also help proxies to make decisions that better reflect the preference and wishes of the person they represent [32, 51].

### Paternalistic attitudes to protection from research

Ensuring the protection of the groups considered most vulnerable to potential harm is an important aim of research ethics, and also those with professional responsibility to care for groups unable to protect their interests through informed consent. However, our survey of health and social care professionals, which explored their knowledge about the legislation governing research involving adults lacking capacity, revealed a deeper and more worrying issue about their attitudes towards the inclusion of people with impaired decision-making capacity in medical research [29]. A number of participants stated that people who lack capacity should never be included in research, both from an ethical and a legal perspective, and some voiced concerns that only the person’s medical practitioner or an appointed judge should have the authority to decide about research participation, and only when it is determined to be in their best interests to take part [29]. There was a widespread lack of understanding that the decision about research participation should not be based on a determination of best interests [52] and should instead be based on what the person’s wishes and feelings about taking part would be, which can only be determined through consulting with those that know them well. This paternalistic approach, which positions health and social care professionals as the ‘gatekeepers’ to research [53], is often reported anecdotally and creates an additional barrier to research for those who would have wanted to participate and those conducting inclusive trials. There is a need for a sea change in attitudes and greater understanding that people who lack capacity should have equitable opportunities to be able to access research studies and to contribute to scientific knowledge in order for them to receive evidence-based care [53].

### Methodological challenges

Trials involving adults lacking capacity can be methodologically challenging; challenges include the difficulties of developing (or adapting) complex interventions [54], evaluating interventions [55] (including developing a common protocol for use in different jurisdictions [39]), the lack of appropriate outcome measures, and the challenges of ensuring intervention fidelity [17]. However, the frequent exclusion of this group from research has led to Age UK describing them as living in a ‘knowledge shadow’ [56]. There is an increasing focus on using an equity lens to critically review the development and evaluation of health (and other) interventions for populations who may experience health inequities to ensure that the evidence generated is of relevance for both clinical practice and public policy making [57]. The lack of equity in trials of interventions, such as rehabilitation interventions after hip fracture, for adults who lack capacity has been identified as a result of their exclusion from trials [19] based on equity factors (e.g. PROGRESS+ factors [57]). This is due in part to the challenges of conducting trials in settings where there is a high prevalence of impaired capacity, and which may be in settings where research is rarely conducted [13, 19, 27, 28]. This may be further compounded by the challenges raised by a lack of validated outcome measures for use in trials involving adults lacking capacity (for example, there are few mental health measures for people with severe and profound intellectual disabilities [58]) and the feasibility of using existing outcome measurement tools [19]. Cognitive impairment may mean that collecting self-reported outcomes, such as quality of life measures such as the five-level EuroQol five dimensions questionnaire, is challenging in some groups such as care home residents [59]. While work is ongoing to establish the reliability of proxy-reported versions of some such measures [59], the limited range of appropriate and validated measures in these populations will inevitably limit the ability to evaluate the effectiveness (and cost effectiveness) of interventions.

### Lack of ‘person-centred’ approaches in trials

The disconnect between established approaches to providing care for populations who lack capacity, and approaches to the design and conduct of trials involving them, creates a conflict between the care and clinical trial paradigms that acts as a barrier to their inclusion [60]. Person-centred approaches to health care focus on flexibility and relationality through treating people as individuals, respecting their rights as persons, building mutual trust and understanding, and developing relationships [61]. In stark contrast, there is little explicit attention paid to the concept of person-centredness in trials involving these same populations [62]. Researchers conducting trials with adults with intellectual disabilities and/or autism identified the need to embrace a range of person-centred approaches, particularly around consent processes, to ensure trials are more inclusive [60]. Adopting these approaches, particularly the tailoring of information provision and consent processes, the use of decision aids and visual aids to improve cognitive accessibility, and revisiting consent after data collection, are key to successful trials involving adults lacking capacity [60]. A framework for person-centred research has been proposed that aims to move beyond traditional research governance approaches to consent which focuses on the ‘protection of vulnerable people’ and the signing of a consent form [62]. Long established practices such as person-centred approaches to inclusionary consent with people living with dementia [63], and inclusive approaches to accessible information and supportive communication widely used with people with learning disabilities, should be adopted. However, person-centred approaches necessitate long-term and sustained involvement from researchers [62], which requires additional resources and greater recognition that simplified accrual-based metrics may act as a barrier to recruiting participants with more complex cognitive and communication needs. It may also require a flexible approach to the way participation is negotiated and data are collected [62]. While person-centred principles may be challenging to integrate with the methodological rigor required when conducting randomised controlled trials, novel approaches to developing person-centred and randomized controlled trials are in the early stages of development [64] and may warrant further attention.

## Conclusions

Adults lacking capacity to consent are frequently excluded from trials, which impacts on the generalisability of the results to these groups and denies them the opportunity to participate and to benefit from evidence generated through trials. In part, this exclusion is a result of overly restrictive eligibility criteria, which has received the greatest attention from funders; however, a number of systemic and structural barriers to the inclusion of adults who lack capacity have also been identified. Addressing these complex issues will be vital if this under-represented and underserved group are to receive evidence-based interventions and care that are available to other population groups. As this is the first attempt at describing the known barriers to their participation, the barriers described here do not form an exhaustive list, nor are the categories to which they have been assigned (methodological, systemic and structural) considered absolute. Further work to develop this conceptualisation of under-representation in trials for such populations should be considered.

## Data Availability

Not applicable.

## Abbreviations

CTIMP: Clinical trial of investigational medicinal product
NIHR: National Institute for Health Research
REC: Research ethics committee
